# The effect of an 8-week mental training program on ITN test performances of tennis players

**DOI:** 10.1186/s13102-025-01463-1

**Published:** 2025-12-11

**Authors:** Ilimdar Yalcin, Harun Genc, Alican Bayram, Burcu Sila Sezer, Ozkan Isik, Huseyin Gumus, Dario Novak

**Affiliations:** 1 Faculty of Sports Sciences, Bingol University, Bingol, Türkiye; 2 School of Health & Human Sciences, Department of Health Professions, Indiana University, Indianapolis, USA; 3 Faculty of Sports Sciences, Balıkesir University, Balikesir, Türkiye; 4 Directorate of Sports Sciences Application and Research Center, Balikesir University, Balikesir, Türkiye; 5 Faculty of Sports Sciences, Mersin University, Mersin, Türkiye; 6 Faculty of Kinesiology, University of Zagreb, Zagreb, Croatia; 7 Institute of Sport Science and Innovations, Lithuanian Sports University, Kaunas, Lithuania

**Keywords:** Athletic performance, Tennis, Sport psychology, Mental training, ITN test

## Abstract

**Background:**

The ITN (International Tennis Number) test is frequently applied to determine the general game success of tennis players. Mental training is widely used to improve the athletic performance. In this context, this study aimed to investigate the effect of an 8-week mental training program applied to male tennis players on ITN test performances.

**Method:**

Twenty amateur male tennis players voluntarily participated in this study. This study was conducted with a quasi-experimental design and the participants were randomly divided into Tennis and Tennis + Mental training groups. The Tennis group performed only tennis-specific training for 45 minutes, 2 days a week for 8 weeks. The Tennis + Mental training group performed 45 minutes of mental training (imagery, focus, self-talk, and breath control) before each tennis training session, followed by similar training with the tennis group, and the tennis players’ ITN test performances were measured as pre- and post-tests. IBM SPSS package program was used to analyze the obtained data. Two-way repeated measures ANOVA was used to determine the difference between the ITN test and its components of the Tennis and Tennis + Mental training groups. In addition, the percentage differences of time points according to the groups were calculated with the formula “% Δ = (Pre-test - Post-test) / Pre-test *100”.

**Results:**

There was no statistically significant difference between the ITN test performance of the Tennis and Tennis + Mental training groups. When the measurement times of the groups were compared, it was found that there was a statistically significant difference between the ITN test performances. When group and time interactions in ITN test performances were analyzed between groups, a statistically significant difference was found. According to these results, while the ITN test performance of the Tennis group increased by 6.35%, the ITN test performance of the Tennis + Mental training group increased by 16.51%.

**Conclusion:**

It was determined that the ITN test scores of tennis players increased through both Tennis and Tennis + Mental training, applied for eight weeks. However, it was determined that mental training in addition to tennis training provided a higher level of improvement in ITN test performances.

**Clinical trial number:**

Not applicable.

## Introduction

Nowadays, it is known that athletic performance is not only related to physical strength, but also that psychological factors significantly affect athlete performance. As a matter of fact, in recent years, the importance of mental training programs has been emphasized to enhance athletic performance [1–3]. Thus, mental training programs influence not only cognitive processes but also physical performance. Therefore, there is a relationship between mental training programs and physical performance, and improved mental skills can enhance physical performance [4–7]. One of the sports where mental and physical performance are evaluated together is tennis. This sport involves hitting the ball with a racket over the net into the opponent’s court [8, 9]. The height, speed and angle of the ball sent to the opponent’s court are important to score points by making the opponent make a mistake. Since tennis players’ hitting abilities can be linked to performance, assessing these abilities is important for athletes and coaches. In this context, the ITN (International Tennis Number) test is frequently applied to determine the general game success of tennis players. In this system, athletes are rated between ITN 1 and ITN 10. While ITN 1 represents high-level athletes, ITN 10 represents athletes who are new to tennis [10]. However, it is known that tennis performance relies not only physical abilities, but also on mental and tactical skills [11, 12]. Therefore, tennis can also provide the opportunity for athletes to develop their mental skills in psychological processes. For this reason, the effect of mental training techniques on tennis performance is remarkable [13].

The techniques and strategies athletes use to improve mental processes and enhance performance are defined as “mental training” [14]. Mental training, defined similarly by different authors [15–17], involves developing various mental skills to use one or more mental techniques in the context of learning, performance and/or optimising competitions [18]. In this context, mental strategies may be noteworthy for individuals to achieve an optimal mental state. One such strategy is imagery [19], which involves mentally visualising the movements performed during a tennis competition or training session [20, 21]. This process, which involves visual and kinesthetic systems, is shaped by previous experiences and directly affects motor and sports performance [22–24]. Another strategy that affects tennis performance is focus. This gain, which can be achieved through mental training, allows the athlete to focus on ball control, competition, task or area of interest and to distance themselves from distracting factors [25]. The third application is self-talk. Internal dialogue is the verbal expressions, either explicit or implicit, that an individual directs towards themselves [26]. Internal dialogue, divided into controlled and uncontrolled [27, 28], plays a vital role due to the nature of tennis, which requires quick decision-making and adaptation to the situation [29]. Therefore, it supports the setting of goals along with the athlete’s motivation [30]. Deep and rhythmic breathing exercises, which provide a physiological basis, help maintain control and psychophysiological balance, supporting calmness in athletic performance [14, 31]. In this context, there are studies in the literature that prove the positive effects of breathing exercises [32]. All these mental training techniques collectively contribute to the psychophysiological aspects of tennis performance. It is known that such techniques are used holistically today (stress and anxiety management, recovery, performance enhancement, competition preparation, and post-injury recovery, etc.) [14]. Due to the holistic benefits of mental training, its acceptance continues to increase [33, 34].

This study aimed to examine the effect of mental training programs applied to male tennis players on ITN levels. This study was one of the few longitudinal investigations examining the effect of mental training programs on athlete performance through the ITN test in amateur male tennis players, and it was important in terms of making original contributions to the literature in this field. In addition, through the results of this study, it was aimed to obtain information about how ITN test and mental training can be used together in tennis and how they can increase the performance of athletes, and thus to contribute to the studies and practices related to the field with a better understanding. Within this scope, the following hypotheses have been established:

H_1_: Mental training applied in addition to 8 weeks of tennis training improves the ITN test components of tennis players.

H_2_: Mental training applied in addition to 8 weeks of tennis training improves the ITN test scores of tennis players.

## Method

### Study design

Experimental research examines the effects of independent variables on dependent variables. In the current study, a quasi-experimental design was used to involve interventions applied to pre-existing groups without full randomization [35].

### Calculation of the sample size

**Fig. 1:**
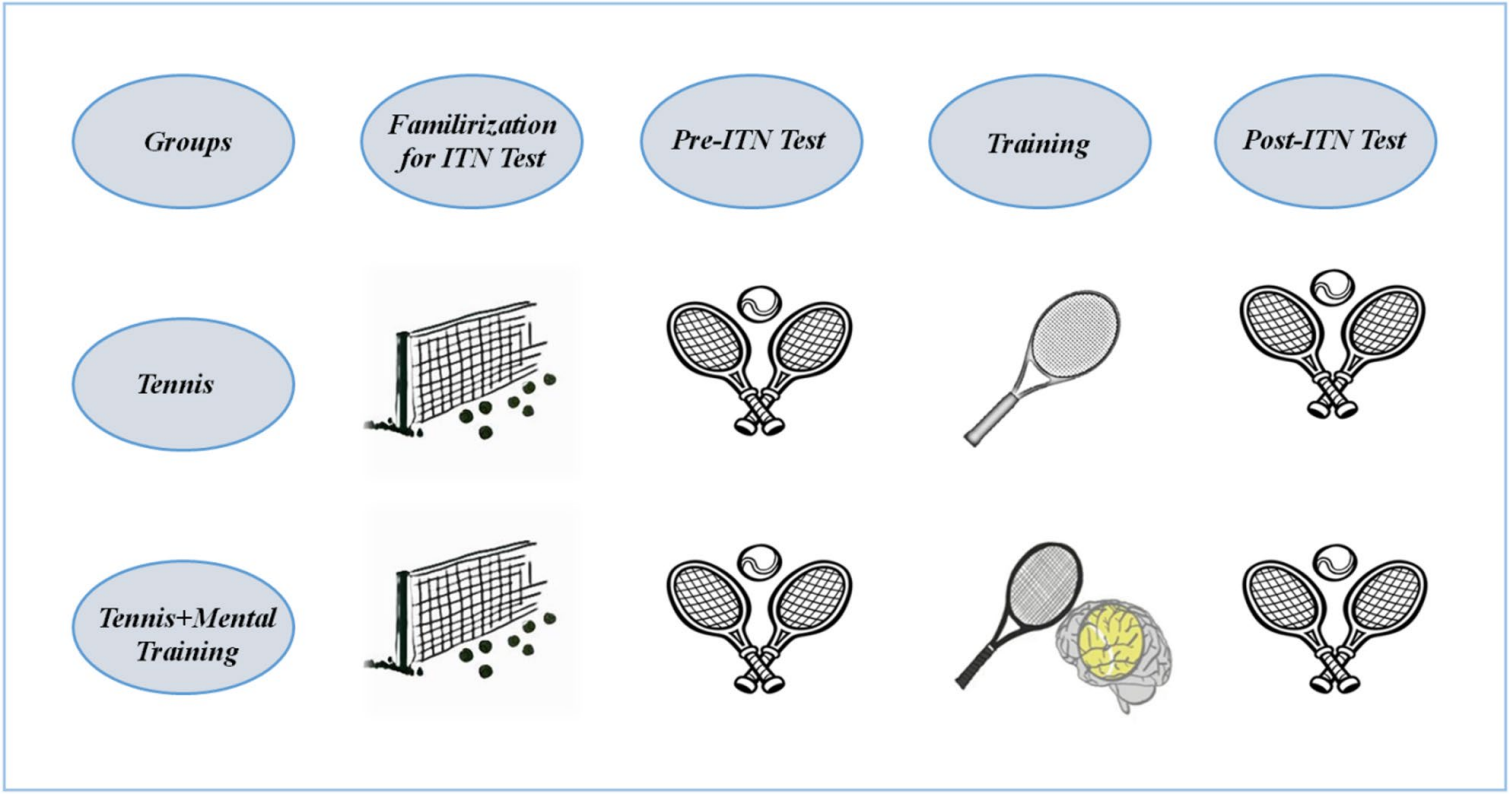
Experimental Design

To determine the required sample size, a power analysis was conducted using G*Power 3.9.1.2 software. It was estimated that a tennis group of 10 participants was needed to detect an effect size (d = 0.86) and a statistically significant difference (α = 0.05; 1-β = 0.80) in ITN test performance before and after 8-week tennis training [10]. Similarly, 10 participants were included in the Tennis + Mental training group to ensure a similar effect size to the tennis group. Therefore, a total of 20 amateur male tennis players aged 18–24 years were included in this study. In the study, tennis players were divided into two groups: Tennis group and Tennis + Mental Training group using a simple random sampling method. The tennis group only applied tennis training protocols for 8 weeks. In the Tennis + Mental training group, both tennis and mental training protocols were applied (Fig. 1).

### Research group and ethical approval

In this study, twenty adult licensed amateur male tennis players who have been playing tennis regularly for at least two years and had ITN ratings of 6–7 participated voluntarily. The true ITN level for a male player is ITN 8. In summary, the participants were at a competitive level. All participants were medically checked before starting the study. Participants were selected from adults who did not have a condition that would prevent them from exercising in the last six months, did not use any chronic medication, and did not have any skeletal-muscular system injuries. It was also determined that the participants had not followed a special diet program or any nutritional supplements in the last six months. They were also warned against drinking alcohol. Moreover, tennis players were asked to sleep adequately before the measurement days. The researchers provided detailed information about the purpose and content of the study to the participants, and all participants provided an informed consent form. This research was approved by the Bingol University Health Sciences Scientific Research and Publication Ethics Board (E-33117789-770−153492), and the research was conducted in accordance with the Declaration of Helsinki.

### Experimental design

In the study, tennis players were informed in detail about the training sessions before starting the study protocol. After the briefing, the ITN test was introduced with familiarization training before starting the pre-test. Participants did not participate in any training program other than the one provided during this study. Before and after the study, ITN tests, one of the most common tests applied by the International Tennis Federation, were applied to measure the performances of tennis players. The details of the ITN test are as follows:

#### Groundstroke depth assessment

Includes a power aspect (10 alternate forehand and backhand ground strokes). The Groundstroke Depth Assessment has been designed to enable players to test their control, depth, and power. Players will receive Double Points if the second bounce is beyond the Bonus Line. Players only receive points for hitting balls into the singles playing area of a tennis court. The player hits 10 balls that are fed to alternate sides, one Forehand, one Backhand, one Forehand, one Backhand, etc. Points are awarded based on where the ball lands on the first and second bounce. When a ball’s first bounce lands anywhere outside the normal singles playing area, it scores zero points.

#### Groundstroke accuracy assessment

Includes a power aspect (6 alternate forehands and backhands down the line and 6 alternative forehands and backhand cross-court). The player should hit each ball down the line. Six balls are fed to alternately (one forehand, one backhand, one forehand, one backhand), etc. The player should hit each ball cross-court. Points are awarded based on where the ball lands on the first and second bounce. When a ball’s first bounce lands anywhere outside the normal singles playing area, it scores zero points.

#### Volley depth assessment

Includes a power aspect (8 alternate forehand and backhand volleys). The player should hit 8 balls that are fed to alternate sides, one Forehand, one Backhand, one Forehand, one Backhand, etc. Points are awarded based on where the ball lands on the first and second bounce. When a ball’s first bounce lands anywhere outside the normal singles playing area, it scores zero points.

#### Serve assessment

Includes a power aspect (12 serves in total, 3 serves in each target area). The player hits 12 serves. Three serves to the wide area of the first service box, three serves to the middle area of the first service box, three serves to the middle area of the second service box and three serves to the wide area of the second service box. Points are awarded based on where the ball lands on the first and second bounce. If the first serve lands anywhere in the correct service box, no second serve is required. If the serve is a let, the serve is replayed. When a ball’s first bounce lands anywhere outside the normal singles playing area, it scores zero points.

#### Mobility assessment

Measures the time it takes a player to pick up five tennis balls and return them individually to a specified zone. The score is recorded in seconds. Points are awarded based on the time it takes to complete this task. The faster a player completes the task, the more points are awarded [36, 37].

### Mental training protocol

The mental training protocol was developed by reviewing the existing literature [38, 39]. Mental training techniques (imagery, focus, self-talk, and breath control) were applied to the athletes using a video-assisted program 2 days a week for 8 weeks, with each session of 45 min. The videos included tennis-specific movements and demonstrations by tennis players. To improve the mental skills of tennis players, a concept introduction and a video-based approach were exhibited in the first week. As the weeks progressed, mental training exercises were expanded and intensified. The details of the 8-week mental training program were shown in Table 1.

**Table 1:** 8-Week mental training protocol

1. Week	Video presentation and demonstration of selected skills
2. Week	Practices for regular breathing and deepening relaxation exercises
3. Week	Deepening relaxation and self-talk exercises
4. Week	Exercises for clarity and concentration on the object in the mind
5. Week	Imagery and self-talk exercises for basic hitting techniques in tennis
6. Week	Watching videos about basic hitting techniques for imagery in tennis
7. Week	Self-talk exercises for volley hitting techniques in tennis
8. Week	ITN service imagery exercises to focus on power and hitting skill

### Tennis training protocol

A tennis training program was applied to the tennis players for 8 weeks, 2 days a week. Each session continued for 45 min, and 10 min of warm-up and cool-down exercises were performed before and after the training. During the training period, apart from the warm-up and cool-down exercises, exercises on basic hitting techniques, volley depth and power stroke, and ITN serve, power, and stability stroke techniques were applied. The details of the 8-week tennis training program were shown in Table 2.

**Table 2:** 8-Week tennis training protocol

1. Week	Warm-up Forehand parallel hitting drills Cool-down
2. Week	Warm-up Forehand cross hitting drills Cool-down
3. Week	Warm-up Backhand parallel hitting drills Cool-down
4. Week	Warm-up Backhand cross hitting drills Cool-down
5. Week	Warm-up Forehand/Backhand parallel hitting drills Cool-down
6. Week	Warm-up Forehand/Backhand paralel/cross (mixed) hitting drills Cool-down
7. Week	Warm-up Volley, depth, accuracy, and serve strokes Tennis match Cool-down
8. Week	Warm-up Tennis match Cool -down

### Statistical analysis

IBM SPSS Statistics 24 software was used to analyze the obtained data. Descriptive statistics of the obtained data were given as mean and standard deviation. Additionally, a two-way repeated measures ANOVA was used to determine the differences between the ITN test scores and its components at the two time points (Pre-test and Post-test) for the Tennis and Tennis + Mental training groups. Moreover, percentage differences of time points according to groups were calculated with the formula “% Δ = (Pre-test - Post-test)/Pre-test *100” [40]. The confidence interval was 95% and values below *p* <.05 were considered statistically significant.

## Results

**Table 3:** The effect of 8-week mental training practices on ITN test components in tennis players

ITN Test Components	Groups	N	Pre-test	Post-test	Total	%Δ	F_(1,18)_	p	η_p_^2^
			$\overline X\pm S.D.$	$\overline{X\pm S.D.}$	$\overline X$± S.D.				
Groundstroke Depth Assessment	Tennis	10	44.00±7.24	47.00±7.15	45.50±7.20	6.82	0.050	0.826	0.634
	Tennis + Mental Training	10	40.30±7.15	49.30±6.90	44.80±7.03	22.33			
	Total	20	42.15±7.26	48.15±6.94			Interaction F_(1,18)_ = 31.154 *p* =.001*; η_p_^2^ = 0.003		
			F_(1,18)_ = 124.615 *p* =.001*; η_p_^2^ = 0.874						
Volley Depth Assessment	Tennis	10	45.00±3.37	47.00±3.43	46.00±3.40	4.44	0.813	0.379	0.043
	Tennis + Mental Training	10	43.20±3.99	51.70±3.89	47.45±3.94	19.68			
	Total	20	44.10±3.71	49.35±4.31			Interaction F_(1,18)_ = 85.449 *p* =.001*; η_p_^2^ = 0.826		
			F_(1,18)_ = 222.978 *p* =.001*; η_p_^2^ = 0.925						
Groundstroke Accuracy Assessment	Tennis	10	45.80±4.08	48.40±4.33	47.10±4.21	5.68	1.969	0.178	0.099
	Tennis + Mental Training	10	45.40±4.53	54.10±4.09	49.75±4.31	19.16			
	Total	20	45.60±4.20	51.25±5.04			Interaction F_(1,18)_ = 148.840 *p* =.001*; η_p_^2^ = 0.892		
			F_(1,18)_ = 510.760 *p* =.001*; η_p_^2^ = 0.966						
Serve Assessment	Tennis	10	40.70±5.25	42.30±5.27	41.50±5.26	3.69	1.817	0.194	0.092
	Tennis + Mental Training	10	41.30±3.09	46.90±3.21	44.50±3.15	13.56			
	Total	20	41.00±4.21	44.60±4.86			Interaction F_(1,18)_ = 97.297 *p* =.001*; η_p_^2^ = 0.844		
			F_(1,18)_ = 315.243 *p* =.001*; η_p_^2^ = 0.946						
Mobility	Tennis	10	40.20±3.88	44.70±4.55	42.50±4.34	-	2.465	134	0.120
	Tennis + Mental Training	10	38.10±4.84	40.70±5.96	39.40±5.40	-			
	Total	20	39.15±4.40	42.70±5.55			Interaction F_(1,18)_ = 0.346 *p* =.937; η_p_^2^ = 0.049		
			F_(1,18)_ = 13.078 *p* =.002*; η_p_^2^ = 0.421						

*p <.05,$\overline X$, Mean *S.D* Standard Deviation, %Δ Percentage difference, *F*_(1,18)_ Degree of Freedom and Error, η_p_^2^: Partial Eta Square

When Table 3 was analyzed, it was determined that there was no statistically significant difference between tennis and Tennis + Mental training groups in ITN test components such as depth, volley, accuracy, serve, and mobility. When the measurement times of the groups were compared, it was found that there was a statistically significant difference between the scores of depth, volley, accuracy, serve, and mobility. When the groups and times interactions of ITN test components were examined, it was found that there was no difference in mobility, but there was a difference in depth, volley, accuracy, and serve. According to these results, both groups showed improvement in mobility over time, but the amount of improvement did not differ between groups. Moreover, the depth, volley, accuracy, and serve scores of the tennis group increased by 6.82%, 4.44%, 5.68% and 3.69%, respectively, at the end of 8 weeks of regular tennis training, while the depth, volley, accuracy, and serve scores of the Tennis + Mental training group increased by 22.33%, 19.68%, 19.16% and 13.56%, respectively. These results show that mental training in addition to 8-week regular tennis training can increase depth, volley, accuracy, and serve scores of ITN test components more than tennis training.

**Table 4:** The effect of 8-week mental training practices on ITN test scores in tennis players

Variable	Groups	N	Pre-test	Post-test	Total	%Δ	F_(1,18)_	p	η_p_^2^
			$\overline X\pm S.D$	$\overline{X\pm S.D.}$	$\overline X\pm S.D.$				
ITN Test Score	Tennis	10	215.70±12.10	229.40±11.85	222.55±11.98	6.35	0.253	0.621	0.007
	Tennis + Mental Training	10	208.30±15.10	242.70±13.83	225.50±14.47	16.51			
	Total	20	212.00±13.85	236.05±14.27			Interaction F_(1,18)_ = 108.784 *p* =.001*; η_p_^2^ = 0.014		
			F_(1,18)_ = 587.376 *p* =.001*; η_p_^2^ = 0.970						

*p <.05,$\overline X$, Mean *S.D*. Standard Deviation, %Δ Percentage difference, *F*_(1,18)_ Degree of Freedom and Error, η_p_^2^ Partial Eta Square

When Table 4 was analyzed, it was found that there was no statistically significant difference between the ITN test scores of the Tennis and Tennis + Mental training groups. When the measurement times of the groups were compared, it was found that there was a statistically significant difference between the ITN test scores. When group and time interactions in ITN test scores were analyzed, it was found that there was a statistically significant difference. According to these results, the ITN test scores of the tennis group increased by 6.35%, while the ITN test scores of the Tennis + Mental training group increased by 16.51%. This result shows that mental training practices in addition to 8-week regular tennis training can increase ITN test scores more than tennis training.

## Discussion

This study aimed to investigate the effects of mental training programs applied to male tennis players on ITN test performance. The main findings of the study revealed no significant difference between male athletes who participated in tennis training and those who also participated in the Tennis + Mental training program. However, when the measurement periods were compared, improvements of 16.51% in the Tennis + Mental Training group and 6.35% in the Tennis group were observed over 8 weeks. This finding suggests that mental training programs added to regular tennis training can be effective in enhancing the ITN test performance of tennis players. The findings align with existing literature that mental training enhances both cognitive and motor performance [41, 42]. Specifically, techniques such as visualization, attention control, self-talk, and breathing regulation contribute to motor development, reduce anxiety, and help maintain focus [43–48]. In fact, in the study by Gonzalez-Fernandes et al. [49], improvements in decision-making, recovery, psychological and physiological endurance parameters were reported. Supporting our findings, Mamassis and Doganis reported that mental training programs increased tennis performance [50]. Contrary to the findings, there were also studies showing that mental training did not affect athletic performance [51, 52]. When the studies reporting that mental training has no effect are examined, it is stated that the participants showed limited progress due to their specialization and the resulting memorization of movements.

According to the secondary findings of the study, significant improvements were observed in all parameters except mobility, one of the ITN test components, when group and time interactions were analyzed. Mobility may not have been affected by mental training since it was evaluated more as a parameter based on physical skills. In fact, both groups showed improvement due to mobility at measurement times, but the amount of improvement did not differ between groups. However, the improvements in ITN test components were 6.82%, 4.44%, 5.68%, and 3.69% and 22.33%, 19.68%, 19.16%, and 13.56% in technical skills such as depth, volley, accuracy, and serve for tennis players in the Tennis group and Tennis + Mental training group, respectively. Therefore, it can be said that tennis training alone has a positive effect on ITN performance [53–56]. These results suggest that mental training applied in addition to tennis training may have a complementary effect on the ITN test components of tennis players. In fact, there are studies supporting this, showing that mental training parameters affect ITN test components such as serve [57–62] depth [63], and volley [64]. These results suggest that mental training techniques should be practiced more effectively by tennis players.

When the measurement times were compared, statistically significant differences were found in depth, volley, accuracy, and serve scores, but not mobility. The development of technical skills may be influenced by mental training due to increased motivation and self-confidence [65]. A systematic review also showed improvements in motor learning and performance among athletes using mental training techniques [64]. Contrary to the findings obtained, there are also studies indicating that mental training practices do not show a change in terms of depth, volley, accuracy, serve, and mobility [24, 36, 52, 66]. It can be said that this difference is due to the evaluation of skills that are difficult or easy to acquire in the same time period.

## Conclusion

Eight weeks of training improved both the ITN test components and ITN test scores in both the Tennis and Tennis + Mental training groups. This suggests that mental training may enhance ITN performance when combined with physical tennis training. The data obtained suggest that training techniques can support the development of tennis-specific technical development. In this context, it suggests that holistically combining mental and physical training can have a positive impact on tennis performance. Overall, the findings suggest that mental and physical training played a complementary role in the current study.

### Limitations

Firstly, selecting only amateur male tennis players limits the generalizability of the results obtained to professional tennis players or tennis players at different levels and gender. In addition, the 8-week training period may be insufficient to evaluate the long-term effects on tennis players. Additionally, the use of only the ITN test performance to determine physical performance in the study may have limited the evaluation of tennis players. The tennis players’ imagery ability is a crucial factor to consider and evaluate when using mental imagery intervention [67]. Indeed, it can influence tennis performance in novice players [68]. It can be assessed by questionnaires such as the Movement Imagery Questionnaire [69] or Likert-type scales. However, unmeasured sensory impact can be measured with scales (e.g., motivation, perceived exertion, etc.). This study was designed as a quasi-experimental. Longitudinal studies with the true experimental study, such as the Solomon four-group model, are needed to provide more detailed results.

### Strengths and weaknesses of the research

When the strengths of the study are examined, it can be said that the combination of sport psychology and training science and its application with a quasi-experimental design will contribute to the field. Furthermore, this study will provide a strong basis for coaches and sports scientists to integrate mental training techniques into tennis programs. Additionally, using an objective measurement method (ITN test) may increase the reliability of the findings. In addition, the study had certain weaknesses. One limitation is that the sample size was relatively small (*n* = 10 per group) and another is that tennis players’ individual differences (stress, motivation, past experiences, etc.) could not be controlled. In addition, it can be considered as a weakness in terms of generalizability to sports branches by examining the research only in the tennis branch.

### Recommendations

Replicating this study with professional players and athletes from different age groups would provide a valuable basis for future research. In addition, long-term effects on tennis players can be examined by increasing the training sessions. In the current study, 8-week mental training techniques were found to have no direct significant effect on ITN test performance of tennis players. However, in future research, adaptations such as longer training programs may lead to improvements in this performance domain. In addition, testing different mental training intensities and incorporating additional cognitive performance measures may broaden the scope of future research.

## Data Availability

The data sets used and/or analyzed during the current study are available from the corresponding author on reasonable request.
